# Supplementary material for: Ontology-driven weak supervision for clinical entity classification in electronic health records
Source: ArXiv. 2020 Aug 5:arXiv:2008.01972v2. Originally published 2020 Aug 5. Preprint. [Version 2] (PMC7418750)
Supplement: 1 [file NIHPP2008.01972V2-supplement-1.pdf]

Title

Ontology-driven weak supervision for clinical entity classification in electronic health records

Supplementary Information

Supplementary Figures

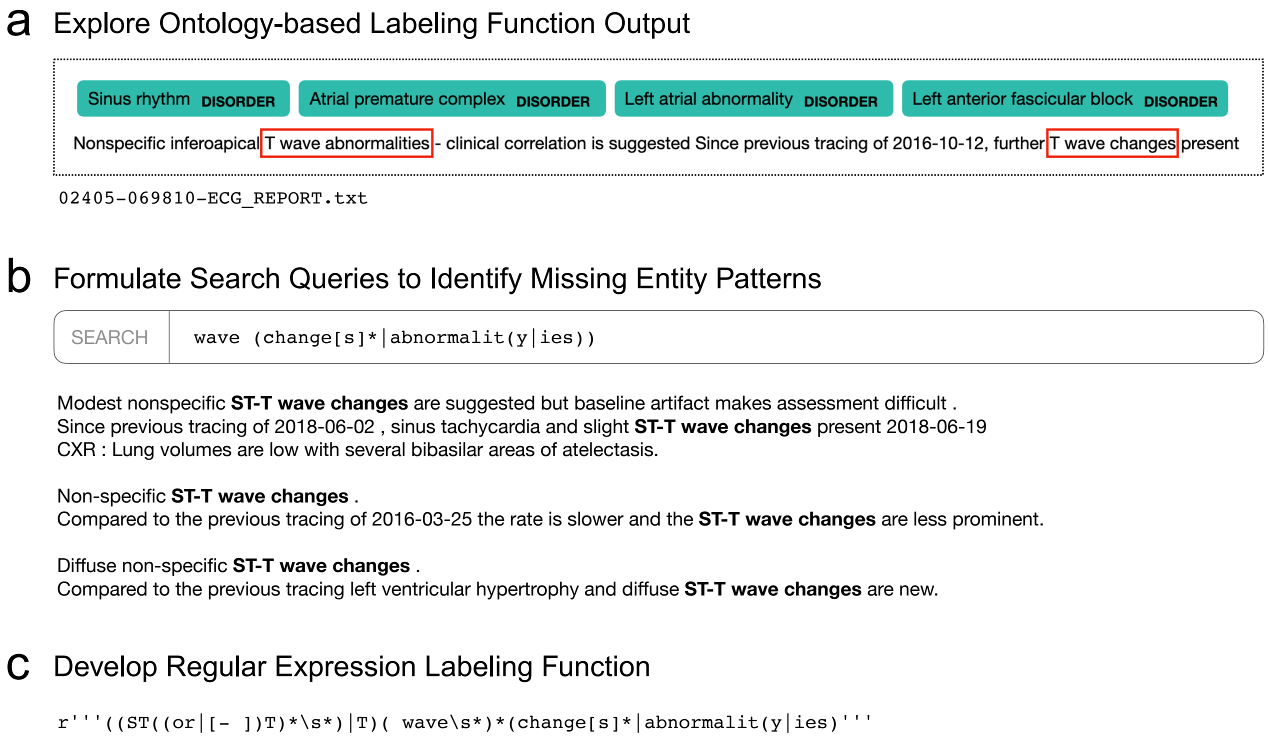

Figure 5: **Example workflow for developing task-specific labeling functions**. **a** Users examine documents tagged by our ontology-based labeling functions and search for common, out-of-ontology entity mentions, in this case "T wave changes" in ECGs. **b** Users create a search query to identify similar missing mention patterns in other documents. **c** Based on the set of documents returned via the search query, users refine their entity pattern into a regular expression which can automatically be used as a labeling function when coupled with a target class label (ShARe/CLEF 2014 disorders in this example).

```
# load semantic types and specify entity mapping
entity_classes = {
   "Antibiotic"    :1,
   "Clinical Drug" :1
}

# define entity categories with classes y \in {0,1}
categories = {
   name:0 if name not in entity_classes else entity_classes[name]
   for name in umls.semantic_types()
}

# build ontology (t \rightarrow [p_1,..., p_k]) and synsets ({\hat{t}_1,...,\hat{t}_n})
ontology = build_entity_map(umls["SNOMEDCT_US"], categories)
synsets  = build_synset_map(umls["SNOMEDCT_US"], categories)

# labeling functions
lfs = [
   SemanticTypeLabelingFunction(name="LF_SNOMED", ontology),
   SynSetLabelingFunction(name="LF_SNOMED_synsets", synsets)
]
```

Figure 6: **Example ontology-based labeling functions.** Semantic type and synset labeling functions do not require that users manually code rules, only that they specify ontologies with sufficient coverage for an entity class of interest. These examples initialize labeling functions for a simple definition of “drug” using the SNOMEDCT_US terminology from the UMLS.

```
rgxs = [
    r"(ACEi|ACE inhibitor[s]*)",
    r"([l][- ](glutathione|arginine))",
    r"([A-Z]){2}[0-9]{3,}",
    r"((alpha|beta|gamma)[-][T])"
]
lf = RegexLabelingFunction(name="LF_chemicals_rgx", rgxs=rgxs, label=1))


rgxs = [
    r"\b([A-Za-z0-9]+?[rlntd]ase[s]*)\b",
    r"[A-Za-z0-9]+ factor[s]*",
    r"\b(anti[a-z]+)\b"
]
lf = RegexLabelingFunction(name="LF_not_chemicals_rgx", rgxs=rgxs, label=0))
```

Figure 7: **Example task-specific labeling functions.** Regular expression labeling functions are developed by manually inspecting unlabeled data and identifying common patterns for the entity of interest. These examples are for chemical tagging in B5CDR.

Supplementary Tables

Task	Method	Ablation Tier	Precision	Recall	F1
Chemical	MV	Guidelines	90.7 ±0.0	3.1 ±0.0	6.0 ±0.0
Chemical	MV	Guidelines+UMLS	87.0 ±0.0	76.8 ±0.0	81.6 ±0.0
Chemical	MV	Guidelines+UMLS+Other	74.6 ±0.0	85.7 ±0.0	79.8 ±0.0
Chemical	MV	Guidelines+UMLS+Other+Rules	78.3 ±0.0	84.2 ±0.0	81.1 ±0.0
Chemical	LM	Guidelines	90.7 ±0.0	3.1 ±0.0	6.0 ±0.0
Chemical	LM	Guidelines+UMLS	89.0 ±0.2	82.3 ±0.2	85.5 ±0.1
Chemical	LM	Guidelines+UMLS+Other	91.0 ±0.2	85.2 ±0.2	88.0 ±0.1
Chemical	LM	Guidelines+UMLS+Other+Rules	90.8 ±0.4	87.7 ±0.4	89.2 ±0.2
Chemical	WS	Guidelines	76.0 ±6.7	7.8 ±3.1	14.0 ±5.0
Chemical	WS	Guidelines+UMLS	87.0 ±0.1	84.6 ±0.2	85.8 ±0.1
Chemical	WS	Guidelines+UMLS+Other	85.7 ±0.3	91.5 ±0.2	88.5 ±0.2
Chemical	WS	Guidelines+UMLS+Other+Rules	91.0 ±0.4	91.2 ±0.3	91.1 ±0.1
Chemical	FS	Supervised	92.1 ±0.4	92.6 ±0.7	92.4 ±0.2

Table 5: Complete performance metrics for BC5CDR chemical tagging for all supervision tiers. Scores are the mean and ±1 SD of 5 random weight initializations.

Task	Method	Ablation Tier	Precision	Recall	F1
Disease	MV	Guidelines	58.5 ±0.0	6.8 ±0.0	12.3 ±0.0
Disease	MV	Guidelines+UMLS	67.8 ±0.0	65.2 ±0.0	66.5 ±0.0
Disease	MV	Guidelines+UMLS+Other	71.9 ±0.0	77.8 ±0.0	74.7 ±0.0
Disease	MV	Guidelines+UMLS+Other+Rules	74.1 ±0.0	78.7 ±0.0	76.4 ±0.0
Disease	LM	Guidelines	58.5 ±0.0	6.8 ±0.0	12.3 ±0.0
Disease	LM	Guidelines+UMLS	70.8 ±0.9	71.3 ±0.1	71.0 ±0.4
Disease	LM	Guidelines+UMLS+Other	80.9 ±0.9	77.0 ±0.7	78.9 ±0.1
Disease	LM	Guidelines+UMLS+Other+Rules	81.8 ±1.1	78.0 ±0.7	79.8 ±0.3
Disease	WS	Guidelines	40.9 ±6.8	51.9 ±4.8	45.1 ±3.1
Disease	WS	Guidelines+UMLS	69.4 ±0.4	75.2 ±0.4	72.1 ±0.4
Disease	WS	Guidelines+UMLS+Other	76.9 ±0.4	79.7 ±0.3	78.3 ±0.2
Disease	WS	Guidelines+UMLS+Other+Rules	78.0 ±0.4	81.9 ±0.1	79.9 ±0.2
Disease	FS	Supervised	82.6 ±0.4	86.5 ±0.2	84.5 ±0.2

Table 6: Complete performance metrics for BC5CDR disease tagging for all supervision tiers. Scores are the mean and ±1 SD of 5 random weight initializations.

Task	Method	Ablation Tier	Precision	Recall	F1
Disorder	MV	Guidelines	69.2 ±0.0	3.8 ±0.0	7.2 ±0.0
Disorder	MV	Guidelines+UMLS	76.1 ±0.0	57.8 ±0.0	65.7 ±0.0
Disorder	MV	Guidelines+UMLS+Other	74.2 ±0.0	62.4 ±0.0	67.8 ±0.0
Disorder	MV	Guidelines+UMLS+Other+Rules	77.0 ±0.0	66.3 ±0.0	71.2 ±0.0
Disorder	LM	Guidelines	69.2 ±0.0	3.8 ±0.0	7.2 ±0.0
Disorder	LM	Guidelines+UMLS	73.2 ±0.0	61.6 ±0.0	66.9 ±0.0
Disorder	LM	Guidelines+UMLS+Other	74.1 ±1.4	63.3 ±0.5	68.3 ±0.3
Disorder	LM	Guidelines+UMLS+Other+Rules	79.4 ±0.8	71.1 ±0.4	75.0 ±0.2
Disorder	WS	Guidelines	35.0 ±5.0	53.9 ±5.5	41.9 ±2.7
Disorder	WS	Guidelines+UMLS	74.1 ±0.3	64.8 ±0.5	69.1 ±0.3
Disorder	WS	Guidelines+UMLS+Other	70.8 ±0.2	67.5 ±0.3	69.1 ±0.2
Disorder	WS	Guidelines+UMLS+Other+Rules	79.4 ±0.2	73.4 ±0.3	76.3 ±0.1
Disorder	FS	Supervised	77.7 ±0.5	81.7 ±0.1	79.6 ±0.3

Table 7: Complete performance metrics for ShARe/CLEF 2014 disorder tagging for all supervision tiers. Scores are the mean and ±1 SD of 5 random weight initializations.

Task	Method	Ablation Tier	Precision	Recall	F1
Drug	MV	Guidelines	76.2 ±0.0	14.8 ±0.0	24.8 ±0.0
Drug	MV	Guidelines+UMLS	70.1 ±0.0	81.9 ±0.0	75.5 ±0.0
Drug	MV	Guidelines+UMLS+Other	69.5 ±0.0	82.0 ±0.0	75.3 ±0.0
Drug	MV	Guidelines+UMLS+Other+Rules	81.6 ±0.0	82.9 ±0.0	82.2 ±0.0
Drug	LM	Guidelines	77.5 ±0.0	15.0 ±0.0	25.2 ±0.0
Drug	LM	Guidelines+UMLS	75.5 ±0.1	79.7 ±0.0	77.5 ±0.1
Drug	LM	Guidelines+UMLS+Other	75.9 ±0.1	81.5 ±0.2	78.6 ±0.1
Drug	LM	Guidelines+UMLS+Other+Rules	86.2 ±0.3	85.4 ±0.7	85.8 ±0.4
Drug	WS	Guidelines	30.0 ±5.9	83.0 ±1.0	43.7 ±6.2
Drug	WS	Guidelines+UMLS	72.6 ±0.3	83.5 ±0.1	77.7 ±0.2
Drug	WS	Guidelines+UMLS+Other	75.7 ±0.2	83.0 ±0.3	79.2 ±0.2
Drug	WS	Guidelines+UMLS+Other+Rules	88.1 ±0.2	88.5 ±0.3	88.3 ±0.3
Drug	FS	Supervised	93.7 ±0.3	92.7 ±0.4	93.2 ±0.3

Table 8: Complete performance metrics for i2b2/n2c2 2009 drug tagging for all supervision tiers. Scores are the mean and ±1 SD of 5 random weight initializations.

Parameter	Values
learning rate	[0.01, 0.005, 0.001, 0.0001]
l2	[0.001, 0.0001]
epochs	[50, 100, 200, 600, 700, 1000]
precision init	[0.6, 0.7, 0.8, 0.9]

Table 9: Label model hyperparameter grid.

Parameter	Values
learning rate	[5e-5, 1e-5, 1e-3]
epochs	[5, 25, 50, 100]

Table 10: BioBERT hyperparameter grid.

Supplementary Note

Task-specific Rule Design: After using Trove to combine multiple ontologies to label entities, we often want to incorporate additional supervision signal to capture more out-of-ontology entities and further improve classification performance. While any existing rule-based system can be used as a labeling functions, either treated as a gestalt, black box labeler or broken down into more modular rules, in this work we largely focus on regular expression labeling functions. Regular expressions are flexible, map to a simple supervision paradigm where users are writing search queries, and correspond to how many rule-based systems are designed in practice [45].

In Supplementary Fig. 5 we illustrate an example workflow for developing a labeling pattern which relies on a mix of data exploration and writing search queries. We assume all documents are queryable via a search index backend such as Elasticsearch [**?**]. First, a user browses a random sample of notes to identify common missing or incorrect entity spans, as generated by our initial ontology-based labeling functions. Second, once a target set of missing entities is identified, the user creates a search query to find similar entity mentions, e.g., "ST-T wave changes" in the example below. Finally, the set of retrieved results is used to expand upon a set of regular expressions, which is then mapped to a class label for use as a labeling function.

Since labeling functions consisting of a single pattern generally have low coverage and often low conflict among other labelers, we typically bundle multiple, related regular expressions into a single labeling function to increase coverage. This process is repeated until the overall label model performance reaches a target performance threshold.

Additional dataset preprocessing: For the DocRelaTime and Negation tasks, labeling functions assume access to explicit datetime mentions (TIMEX3) and clinical event entities (e.g. disorders, drugs, procedures). However, our experiments assume machine-learning based entity taggers are not available for these subtasks. Instead, we use a dictionary of clinical events derived from the UMLS to tag possible event entities, which are used to generate noisy candidate entities for both Negation and DocRelaTime tasks. TIMEX3 entities are tagged using regular expressions and normalized into abstractions supporting datetime math. Labeling functions are applied to these candidates to train the label model, with the resulting probabilistic labels used to train our BioBERT models. For the ShARe/CLEF tasks we report scores on a subset of the overall disorder entity set, removing non-contiguous, relational-style disorders spans, which comprised 7.9% (628) of test set mentions.

Guideline annotation examples: These examples are provided directly in annotation guideline documents.

- Chemical (BioCreative V CDR Task - Data Annotation Guidelines)
  - Positive [ATP, Ca, DCE, Fe, K, Li, NO, O2, amino acid, angiotensin II, angiotensin ii, antidepressant, antidepressant drug, antidepressive agent, cAMP, carbidopa, estrogen, estrogen receptor agonist, estrogenic agent, estrogenic compound, estrogenic effect, ethanolic extract of daucus carota seed, fatty acid, glucose, grape seed proanthocyanidin extract, levodopa, low-dose oral contraceptive, nitric oxide, oral contraceptive, phasic oral contraceptive, polyethylene glycol, saturated fatty acid, steroid, sucrose, thymoanaleptics, thymoleptics]
  - Negative [DNA, adrenergic, anti-HIV agent, anticholinesterase drug, anticoagulant, anticonvulsant, antipsychotic, atom, cellulose, collagen, glucagon, glucocorticoid, glycogen, gold standard, insulin, ion, juice, lipid, lipopolysaccharide, mRNA, molecular, muscarinic, nucleic acid polymer, oligosaccharide, opiate, opioid, opioid alkaloids, opium poppy plant, papaver somniferum, polypeptide, polysaccharide, prolactin, protein, purinergic, saline, starch, water]
- Disease (BioCreative V CDR Task - Data Annotation Guidelines)

- Positive [akathisis, auditory toxicity, bone marrow oedema, cancer, cardiac toxicity, death, dyskinesia, erythroblastocytopenia, hepatitis, hypertension, hypertensive, liver toxicity, ototoxicity, ovarian and peritoneal cancer, pain, partial seizures, peritoneal cancer, toxicity, tumor, visual toxicity]
- Negative [cancerogenesis, complication, deficiencies, deficiency, disease, syndrome, tumorigenesis]

- Disorder (ShARe/CLEF eHealth 2013 Shared Task: Guidelines for the Annotation of Disorders in Clinical Notes)
  - Positive [bowel obstruction, chest pain, chronic gingivitis, colon cancer, crohn, facial droop, lower extremity DVT, lupus, numbness, pain, rash, schizophrenia, severe pre-eclampsia, small bowel obstruction, stroke, tumor, tumor of the skin, watering of the eye]
  - Negative NONE
- Drug (i2b2 Medication Extraction Challenge Preliminary Annotation Guidelines)
  - Positive [CITALOPRAM HYDROBROMIDE, CZI, ECASA, ECASA (ASPIRIN ENTERIC COATED), IV fluid, KCL IMMEDIATE REL, LISINOPRIL, NIFEREX TABLET, NITROGLYCERIN 1/150, NTG, POTASSIUM CHLORIDE, TPN, TYLENOL (ACETAMINOPHEN), TYLENOL (ACETAMINOPHEN), acetaminophen, asa, aspirin, atenolol, avapro, bb, caltrate plus D, caltrate plus D, novolog, diuretic, diuretics, fasting lipids sent, fluocinonide 0.5% cream, furosemide, glucophage, lasix, lasix, lasix, long acting nitrate, nephrotoxic meds, plavix, red blood cells, saline, saline solution, this medication, total parenteral nutrition, tylenol, tylenol 3, nitroglycerin 1/150, vitamin A, vitamin C, vitamin D, vitamin E, vitamin E, vitamins, vitamins A, vitamins C, vitamins D, vitamins E]
  - Negative NONE
